# Zebra finches are able to learn affixation-like patterns

**DOI:** 10.1007/s10071-015-0913-x

**Published:** 2015-08-22

**Authors:** Jiani Chen, Naomi Jansen, Carel ten Cate

**Affiliations:** Behavioural Biology, Sylvius Laboratory, Institute of Biology Leiden, Leiden University, P.O. Box 9505, 2300 RA Leiden, The Netherlands; Leiden Institute for Brain and Cognition, Leiden University, Leiden, The Netherlands

**Keywords:** Affixation, Language evolution, Cognitive asymmetry, Songbird

## Abstract

Adding an affix to transform a word is common across the world languages, with the edges of words more likely to carry out such a function. However, detecting affixation patterns is also observed in learning tasks outside the domain of language, suggesting that the underlying mechanism from which affixation patterns have arisen may not be language or even human specific. We addressed whether a songbird, the zebra finch, is able to discriminate between, and generalize, affixation-like patterns. Zebra finches were trained and tested in a Go/Nogo paradigm to discriminate artificial song element sequences resembling prefixed and suffixed ‘words.’ The ‘stems’ of the ‘words,’ consisted of different combinations of a triplet of song elements, to which a fourth element was added as either a ‘prefix’ or a ‘suffix.’ After training, the birds were tested with novel stems, consisting of either rearranged familiar element types or novel element types. The birds were able to generalize the affixation patterns to novel stems with both familiar and novel element types. Hence, the discrimination resulting from the training was not based on memorization of individual stimuli, but on a shared property among Go or Nogo stimuli, i.e., affixation patterns. Remarkably, birds trained with suffixation as Go pattern showed clear evidence of using both prefix and suffix, while those trained with the prefix as the Go stimulus used primarily the prefix. This finding illustrates that an asymmetry in attending to different affixations is not restricted to human languages.

## Introduction

Language is a uniquely human trait, which makes it a challenge to understand how different components of the language faculty have evolved. One window to provide insights and hypotheses about their origins is by comparative studies on the cognitive abilities of non-human animals (Fitch 2010; Hauser et al. 2002). Such studies can be directed at phylogenetically related taxa, like apes and monkeys. Alternatively, one can examine the presence of relevant cognitive abilities in more distantly related groups in which relatively complex and structured vocalizations evolved independently. One such a group is songbirds. Songbirds show striking cognitive, neural and genetic similarities with humans in vocal perception, production and auditory–vocal learning (e.g., Bolhuis and Everaert 2013; Bolhuis et al. 2010; Doupe and Kuhl 1999; Kriengwatana et al. 2015; Ohms et al. 2010; ten Cate 2014; ten Cate and Okanoya 2012). For this reason, they are excellent model species to explore cognitive abilities that might have been at the basis of language evolution. In the current study, we also use a songbird species, the zebra finch, to examine whether it is able to categorize strings of acoustic elements based on ‘affixation’-like patterns.

Among the components of language, one of the most notable aspects is morphological transformation, such as inflectional morphology. Inflection, such as adding an affix to transform and change the meaning of a word, occurs quite often across the languages. For instance, the great majority of English verbs form their past tense by adding the suffix ‘-ed’ to an unchanged stem. Affixes can also be used to make compound words belonging to different categories, such as ‘prosocial’ versus ‘antisocial.’ Knowledge of affixation rules plays an important role in language development (Kuczaj 1977; Mochizuki and Aizawa 2000; Nagy et al. 1993). Interestingly, the edges of the words are more likely to carry out the grammatical functions; an affix in the first position (prefix) or in the last one (suffix) is much more frequent than affixes in other positions (Endress and Hauser 2011; Endress et al. 2009b). This bias is not only observed in languages. Learning in serial memory tasks also showed that the edge positions of a sequence can be recalled more accurately (Endress et al. 2010; Henson 1998, 1999; Hitch et al. 1996; Wright et al. 1985). Also in artificial language learning, participants were found to reliably generalize regularities at the edges but not in the middle of acoustic sequences (Endress and Mehler 2010; Endress et al. 2005). These examples suggest that prefixation and suffixation patterns are relatively easy to learn and that the linguistic edge-based positional learning competence could be based upon what Endress et al. (2009b) called a ‘perceptual and memory primitive,’ a phylogenetically preexisting cognitive mechanism that constrains rule-based learning in language acquisition and may have guided language evolution. If so, it raises the question to which extend the edge-based positional learning competence is shared with non-human animals and whether they can learn affixation patterns.

Studies of sequential memorization in several species of birds and monkeys have shown that, in general, the edge(s) of a sequence can be recalled better (Comins and Gentner 2010; Endress et al. 2010; Orlov et al. 2000; Terrace et al. 2003; Wright et al. 1985). In an artificial language learning experiment, Endress et al. (2010) showed that chimpanzees also encode the edges of sequences better than the other positions in the sequences, similar to adult humans in the same experiment. Such experiments suggest that animals might also have the ability to recognize and learn affixation patterns. This was examined in a pioneering study by Endress et al. (2009a), in which cotton-top tamarin monkeys were exposed to a set of human speech syllables (‘stems’) that were either preceded or followed by the affix syllable ‘shoy.’ When subsequently presented with novel stems, the tamarins discriminated between words starting with shoy as a ‘prefix’ and those that end with the same syllable as a ‘suffix,’ demonstrating that they generalized the underlying affixation rule. Up to now, there is no evidence of such an ability from other animal species. Given the above-mentioned similarities between songbirds and humans in vocal processing and also because birds show evidence of at least some, albeit simple, rule learning when trained and tested with strings of elements that are artificially structured according to different algorithms (e.g., Chen et al. 2015; Comins and Gentner 2014; Gentner et al. 2006; Seki et al. 2013; Spierings et al. 2015; van Heijningen et al. 2013, 2009), they are promising candidates to examine whether they are capable of discriminating among different affixation patterns and to generalize this to novel strings with the same affixations. If they can, this might be an indication that linguistic affixation learning might have arisen from a more wide spread cognitive ability that is not specific to language nor to humans.

In the current study, we trained and tested zebra finches in a Go/Nogo paradigm to discriminate artificial song element sequences resembling prefixed and suffixed ‘words.’ The ‘stems’ of the ‘words’ consisted of different combinations of a triplet of song elements, to which a fourth element was added as either a ‘prefix’ or a ‘suffix.’ After training, the birds were tested with novel stems, consisting of either rearranged familiar element types or novel element types. We do not want to claim that our experiment can demonstrate the presence of the full formal notions of affixations in a non-human animal. It is important to note, for instance, that our ‘stems’ carry no semantic meaning. What our experiment can demonstrate is whether birds are able of using edge-based learning to detect surface transformations similar to different affixation patterns by learning to discriminate strings differing in the presence of a particular element either before a string (prefix) or after the same string (suffix). Rather than using a habituation paradigm as used in the tamarin study (Endress et al. 2009a), we use a Go/NoGo paradigm. The habituation paradigm can tell whether animals spontaneously detect a change in a pattern, but detecting such a change is not linked to any consequence. The Go/Nogo not only tests whether the animals detect a difference, but also tests whether they can link this to a difference in consequences, analogous to human infants that have to learn over time how different affixations alter word meanings. We also examine whether zebra finches can learn both prefixation and suffixation patterns equally well, or are more sensitive to one or the other type, as has been suggested for human languages (Cutler et al. 1985; Dryer 2005; St Clair et al. 2009). Our results show that the zebra finches are able to learn both regularities. Remarkably, birds that had been trained with prefixation as Go pattern used predominantly the prefix to make their discrimination, while birds trained with suffixation as Go pattern used both prefix and suffix.

## Materials and methods

### Subject and apparatus

Twelve zebra finches (six males and six females) from Leiden University breeding colony were trained and tested individually in sound attenuated chambers. None of the birds had previous experience with any kind of experiment. Six birds participated in Experiment 1; all 12 participated in Experiment 2. The experiment was conducted by using Go/Nogo paradigm in an operant conditioning cage described earlier (van Heijningen et al. 2013). A fluorescent tube on the top of the box emitted daylight spectrum light on a 13.5-L: 10.5-day schedule. Upon pecking a response key, a sound was played through a loudspeaker, attached above the box, at approximately 70 dB. Subjects gained access to food for 10 s after they responded with pecking a second key upon hearing a Go sound. Conversely, if subjects responded to playback of a Nogo sound, the light of the chamber was switched off for 15 s to indicate the error. The second key was active only after the full sound was played.

### Stimuli

**Fig. 1:**
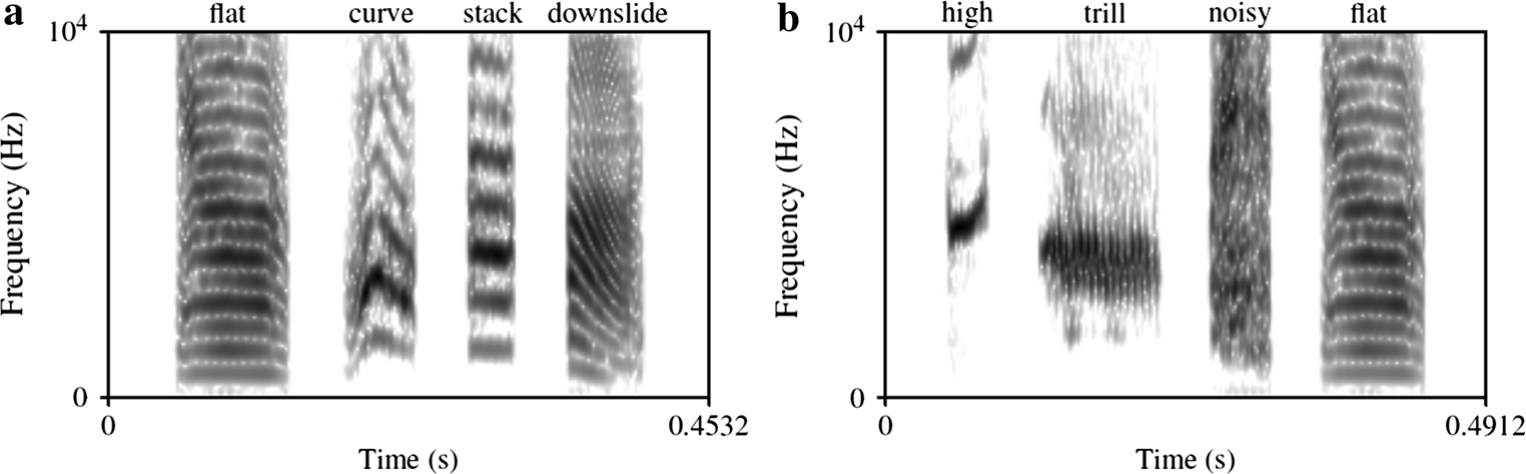
**a**, **b** Spectrograms of GABC stimuli for two different birds

The ‘words’ used in this study consisted of artificially edited sequences consisting of four song elements. These elements were obtained from natural zebra finch songs (undirected songs) originating from our zebra finch song database. Seven elements, ‘flat,’ ‘stack,’ ‘trill,’ ‘downslide,’ ‘high,’ ‘curve’ and ‘noisy’ (see Fig. 1 for examples), were selected based on optimal discriminability. They were ramped and RMS equalized.

Two types of regularities, prefixation and suffixation, were used to construct the stimuli (Table 1). The ‘stems’ of the training stimuli were triplets constructed from three different element types ‘A, B and C’ in different combinations. Each element type occurred in every possible slot over the triplets. A fourth element type ‘G’ was only used as either a ‘prefix’ or a ‘suffix.’ To eliminate pseudo-effects due to an arbitrary parameter of the sounds, the element assignments were shuffled for the subjects; for instance, element ‘A’ could be ‘curve’ for one bird and ‘trill’ for another bird (Fig. 1). In Experiment 1, birds were first trained with three Go and three Nogo stimuli, each consisting of different combinations of an A, B and C element, and either preceded or followed by the affix G. The test stimuli of Experiment 1 were constructed by rearranging the element combinations of the stems. In Experiment 2, the training set included the stimuli for the training as well as those used for testing in Experiment 1. Testing occurred with stimuli in which the stems were formed by the novel element types ‘D, E and F’, which never occurred in the training phase.

**Table 1:** Training and test stimuli of Experiment 1 and 2

Condition	Experiment 1 (group 1)			Experiment 2 (group 1 and 2)			
	Training			Training			
1	Go	Nogo	Test	Go	Nogo	Test 1	Test 2
2	Nogo	Go		Nogo	Go		
Stimulus			**G**ACB	**G**ABC	ABC**G**	**G**DEF	
			**G**BAC	**G**BCA	BCA**G**	**G**EFD	
	**G**ABC	ABC**G**	**G**CBA	**G**CAB	CAB**G**	**G**FDE	ABC
	**G**BCA	BCA**G**	ACB**G**	**G**ACB	ACB**G**	DEF**G**	DEF
	**G**CAB	CAB**G**	BAC**G**	**G**BAC	BAC**G**	EFD**G**	
			CBA**G**	**G**CBA	CBA**G**	FDE**G**	

The table shows the stimuli of Experiment 1 and 2. Subjects in Experiment 1 were trained with six stimuli and tested with newly arranged ‘stems’ consisting of familiar element types. Subjects in Experiment 2 were trained with 12 stimuli and tested with new ‘stems’ consisting of novel element types. For half of the birds the prefixation pattern was used as the Go stimulus while the suffixation was used as the Nogo stimulus and vice versa for the other half of the birds

For each stimulus, 40 ms of silence was inserted between consecutive elements and 50 ms of silence was added at the start and the end to avoid acoustic distortions during playback. The training stimuli followed either a prefixation or a suffixation pattern. For half of the birds, the G-prefix predicted Go stimuli and the G-suffix the Nogo stimuli (Table 1, Condition (1)) and vice versa for the other half of the birds (Table 1, Condition (2)). The test stimuli were constructed by adding the G-suffix or G-prefix to novel stems.

### Procedure

To familiarize the birds with the Go/Nogo task, they were first trained to discriminate a natural song (Go stimulus) from a pure tone (Nogo stimulus). When their responses reached the training criterion (>75 % response to Go stimuli and <25 % response to Nogo stimuli) for at least two consecutive days, they were switched to the next phase of training, in which the experimental stimuli were presented.

Experiment 1 tested whether the birds were able to generalize the affixation patterns of the training stimuli when these were presented in combination with novel stems. Six birds (Group 1) were trained with three pairs of stimuli and subsequently tested with another three pairs of stimuli constructed from familiar element types but in novel combinations (Table 1). The tests started after the birds reached the training criterion to every training stimulus for at least three consecutive days.

In the tests, test stimuli were not reinforced to avoid additional learning. Every test contained 40 presentations of each test stimulus. To prevent extinction of the pecking behavior, only 20 % of all stimulus presentations were non-reinforced stimuli (including test stimuli and two training stimuli from the Go and the Nogo sets). The other 80 % of stimulus presentations consisted of the reinforced training stimuli.

Experiment 2 tested whether the zebra finches could generalize the affixation patterns to stems constructed from novel element types. It also addressed whether the discrimination shown in training and test was dependent on the presence of prefix only, suffix only or both. A total of 12 birds were used. Six of these had previously been used in Experiment 1 (Group 1), and another six (Group 2) had not been trained and tested before. The six pairs of Go and NoGo training and test stimuli in Experiment 1 were combined and used as training stimuli (Table 1). After the responses of the birds to every stimulus of the training reached criterion for at least three days, the first test started. Test 1 examined the response to new stimuli with novel stems consisting of novel element types. The second test was given after Test 1, consisting of the ABC and DEF stems without any affix. If the birds learned both prefixation and suffixation patterns, we expected them to respond to these ‘stem-only’ stimuli at an intermediate level compared to their responses to the ‘affix-versions.’ As in Experiment 1, 20 % of the stimuli were test stimuli, which were not reinforced.

## Results

### Experiment 1

**Fig. 2:**
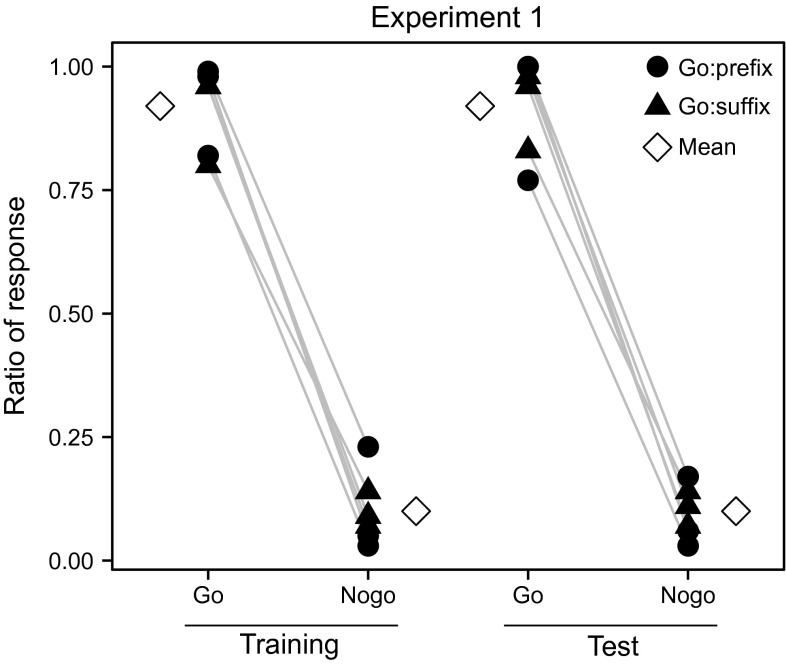
Performances of individual birds of *Experiment* 1. All birds discriminated between prefix and suffix stimuli, both in the training and in the test, and irrespective whether the Go stimulus is a prefix (Go: prefix) or a suffix (Go: suffix). Mean response ratios (the proportion of responses in relation to the number of times a Go-set or a Nogo-set of stimuli has been presented) of all birds are also shown. Both training and test stimuli are constructed with element type A, B and C (in different sequences), using G as affix. Test stimuli are not reinforced; ‘*Go*’ and ‘*Nogo*’ indicate test stimuli that are structurally similar to Go and Nogo training stimuli

All birds (*N* = 6) reached training criterion, on average after performing 2365 trials ± 245 SEM. There was no significant difference between the sexes in their discrimination ratio (DR, calculated as the response to Go stimuli divided by the sum of the response to Go stimuli and the response to Nogo stimuli) (*t* = 1.40, *df* = 4, *P* = 0.234, Student’s *t* test). All birds distinguished non-reinforced test stimuli with different structures as well as they discriminated the reinforced stimuli (Fig. 2). The responding ratios in the test were 0.92 ± 0.04 SEM to the Go pattern and 0.10 ± 0.02 SEM to the Nogo pattern. The DR for individual birds was all higher than 0.5 (DR = 0.908 ± 0.018 SEM), which indicates positive discrimination (Wilcoxon signed-rank test *Z* = −2.201, *P* = 0.028). There was no significant difference between using the prefix or the suffix stimuli as the Go stimuli (responses to the Go pattern: *U* = 3.0, *P* = 0.700; responses to the Nogo pattern: *U* = 3.0, *P* = 0.700, Mann–Whitney *U* test).

### Experiment 2–Test 1

**Fig. 3:**
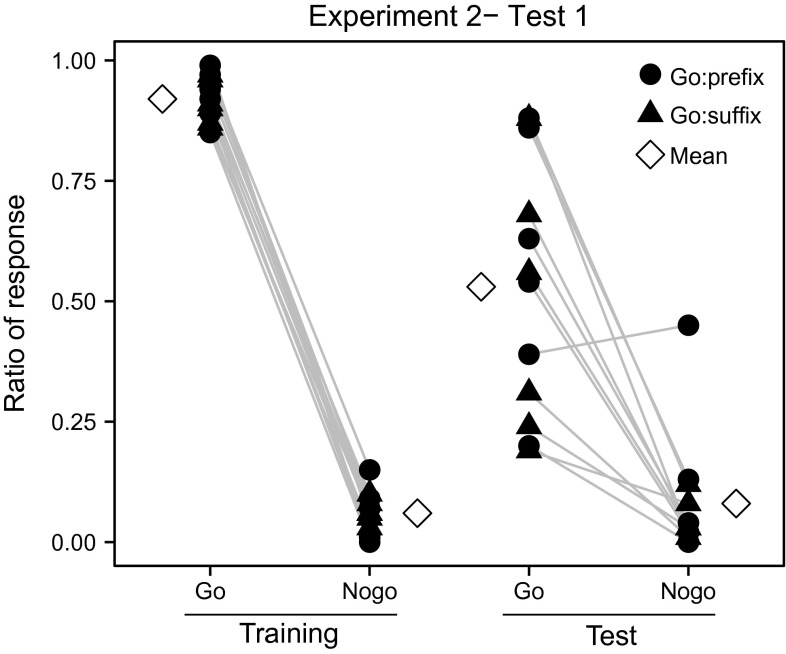
Performance of individual birds in *Experiment* 2, *Test* 1. Eleven birds discriminated between prefix and suffix stimuli in the test irrespective whether the Go stimulus is a prefix (Go: prefix) or a suffix (Go: suffix). Mean response ratios of all birds are also shown. Training stems are constructed with element types A, B and C, while test stems are constructed with element types D, E and F. Element G is used as the affix in both training and test stimuli. Test stimuli are not reinforced; ‘*Go*’ and ‘*Nogo*’ indicate test stimuli that are structurally similar to Go and Nogo training stimuli

All birds (*N* = 12) learned to discriminate the Go and Nogo stimuli in the training. The birds that participated in Experiment 1 (Group 1) maintained the discrimination as soon as they were switched to the training of Experiment 2 (as was to be expected from the test results of Experiment 1). The other six birds (Group 2) reached training criterion after performing 2474 trials ± 454 SEM. No significant difference of DR was found between different sexes (*t* = –0.432, *df* = 10, *P* = 0.675, Student’s *t* test). In Test 1, there was no significant difference between training with a prefix and that with a suffix as Go stimulus (responses to the Go pattern: *U* = 14.50, *P* = 0.589; responses to the Nogo pattern: *U* = 18.0, *P* = 1.0, Mann–Whitney *U* test). The different training groups (Group 1 versus Group 2) also showed no significant difference (responses to the Go pattern: *U* = 13.50, *P* = 0.485; responses to the Nogo pattern: *U* = 15.50, *P* = 0.699, Mann–Whitney *U* test). Therefore, the two groups were pooled. The responding ratios to the test stimuli with affixation patterns similar to Go training stimuli differed significantly from those to test stimuli with affixation patterns similar to the Nogo training stimuli (0.53 ± 0.08 SEM and 0.08 ± 0.04 SEM, respectively (Fig. 3*Z* = −2.934, *P* = 0.003, Wilcoxon signed-rank test). Eleven birds showed a high DR in the test (0.92 ± 0.02 SEM), while one out of the 12 birds did not generalize the Go and Nogo patterns to the test stimuli (DR = 0.47).

### Experiment 2–Test 2

While the previous tests showed no differences in response patterns between training with a prefix and with a suffix as Go stimulus, this test did, therefore, data from the two training conditions are presented separately. Page’s trend test for ordered alternatives (Page 1963; Siegel and Castellan 1981) was applied to detect whether the responses to test stimuli were ordered according to their affixes, testing the hypothesis that the responses to stimuli without an affix are expected to be in between those with a prefix or suffix

### Go: prefix

Responses to the stimulus without affix (ABC and DEF) were compared with their ‘affix-versions’ (GABC and ABCG; GDEF and DEFG).The one bird that did not generalize the Go and Nogo response to stimuli with novel element types was excluded from the test involving the DEF stem.

**Fig. 4:**
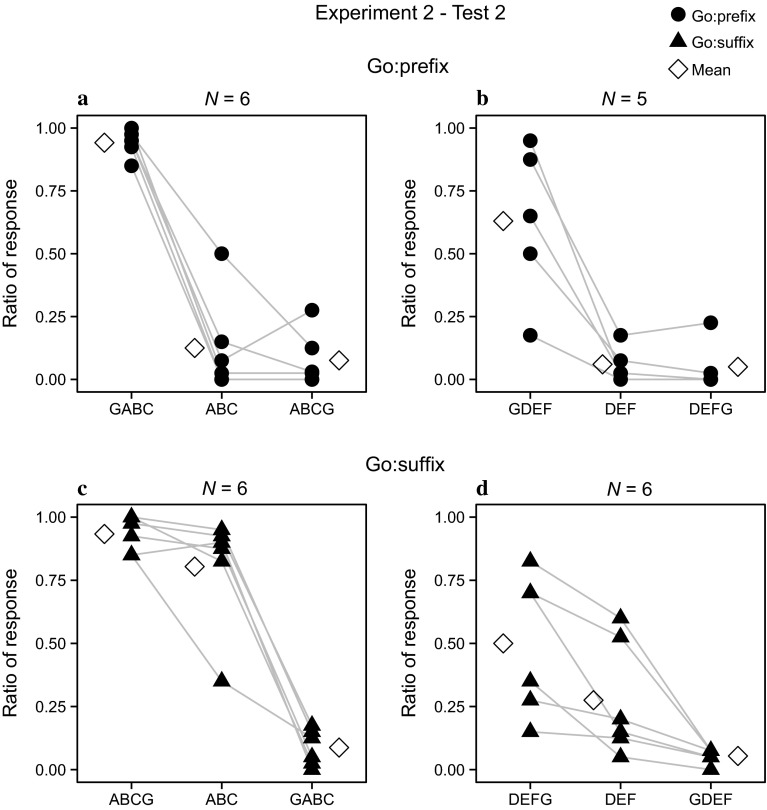
Performance of individual birds in Experiment 2, Test 2. **a** Responses to stimuli with familiar stems for birds trained with the prefix as Go stimulus (Go: prefix). **b** Responses to stimuli with novel stems for birds trained with the prefix as Go stimulus. **c** Responses to stimuli with familiar stems for birds trained with the suffix as Go stimulus (Go: suffix). **d** Responses to stimuli with novel stems for birds trained with the suffix as Go stimulus. Mean response ratios of all birds are also shown

The test showed a significant decline in responses from GABC, ABC to ABCG (*L* = 81.5, *N* = 6, *P* < 0.05, Fig. 4a). However, most birds showed little or no differentiation between ABC and ABCG. Only one bird showed a clear intermediate response to ABC. A similar responding pattern was observed in the test with novel elements. Again a significant decline was found in responses to GDEF, DEF and DEFG (*L* = 68.5, *N* = 5, *P* < 0.05, Fig. 4b). However, the responses to the ‘stem-only’ stimuli were more similar to the responses to suffixed stimuli.

### Go: suffix

In this condition, the training stimuli of the Go pattern ended with a suffix, while the Nogo pattern started with a prefix. The data were analyzed in the same way as above.

The responses to stimuli decreased gradually from the Go pattern, the ‘stem-only’ pattern to the Nogo pattern, whether these consisted of familiar or novel element combinations. Though responses to ABC and ABCG were slightly different among most birds (only one bird showed a clear intermediate response to ABC), there is a significant decline in the responses to ABC when compared to the responses to ABCG. The intermediate response to the stem-only stimulus was shown most clearly in the test with DEF stem (test with ABC stem: *L* = 83, *N* = 6, *P* < 0.05, Fig. 4c; test with DEF stem: *L* = 84, *N* = 6, *P* < 0.05, Fig. 4d).

## Discussion

The results of Experiment 1 showed that the birds perfectly generalized the discrimination obtained during the training to test stimuli that shared the affixes with the training stimuli, but had a novel stem constructed from familiar element types. It shows that the discrimination resulting from the training was not based on memorization of individual stimuli, but on a shared property among Go or Nogo stimuli. This shared property could be having either a G-suffix or G-prefix. However, the result can also be obtained if the birds paid attention to whether the stimuli either started or ended with an A, B or C element. Also, if the birds use the G-element, they can achieve discrimination by attending to either the suffix position only, the prefix position only or both. Experiment 2 addressed these questions. Test 1 shows that discrimination is maintained even when the affixations are connected to stems consisting of novel element types. This discrimination can only be due to attending to the presence and position of the affix: the G-element, and by generalizing the affixation rule to new stems, similar to what has been shown for tamarins by Endress et al. (2009a). So, we conclude that our results provide the first evidence in a non-primate of learning a rule that, at least in its surface pattern, is similar to a linguistic affixation pattern: Birds can identify that presence of a specific vocal unit at one or the other edge of a string is linked to different consequences and generalize this knowledge to novel strings. Our results are also similar to those obtained in an experiment with 9-month-old infants. In this experiment (Gerken 2006), infants were exposed to strings consisting of three CV-syllables. If the exposure strings all ended with the syllable ‘di,’ the infants generalized this pattern to novel stimuli also ending with ‘di.’ Nevertheless, as songbirds are phylogenetically quite distant from humans, our findings should not be taken as evidence that the competence is formally fully similar to that of humans using affixations. As outlined by Berwick et al. (2011), there is quite a gap between the syntactic structures birds use or can detect and those present in human languages. However, our experiment indicates the presence of a processing and generalization competence of affixation-like patterns that is independent of having language. A similar ability may also have been present in pre-linguistic humans and may have acted as a domain-general perceptual and memory primitive (Endress et al. 2009b) that has been co-opted for the evolution of a linguistic phenomenon.

The results of the second test of Experiment 2 demonstrate that birds paid attention to both the prefix and suffix. However, the birds trained with suffixed sequences as Go stimuli responded to the ‘stem-only’ stimuli at a more intermediate level than the birds trained with the prefix as the Go stimulus. This effect was less strong when the stem was ABC. Though the stimulus ABC was not affixed, it overlapped with the first part of the suffixed version used as training stimulus, and birds may have used this as an additional cue to discriminate the stimuli. The test with novel element types excluded the use of such a cue and demonstrated that at least one group attended very clearly to both prefix and suffix (Fig. 4d). All birds were trained with exactly the same stimuli, but the Go and Nogo associations were opposite for the two groups. Birds trained with prefixes as Go stimuli responded strongest to stimuli starting with a ‘G,’ whereas birds trained with the suffix as a Go stimulus showed evidence of using both affixes. It suggests that the responses were guided by both a tendency to pay more attention to the first part of a sequence and paying attention to a shared feature of a stimulus set. For several songbird species, there is evidence that different parts of the song may differ in their information content (e.g., Elfstrom 1990; Kreutzer et al. 1992; Leader et al. 2000; Lengagne et al. 2000; Mundinger 1975; Nelson and Poesel 2007) and, depending on the context, either the beginning or end of songs seems most important to convey particular information. The asymmetry in attending to prefix and suffix as shown by the zebra finches may have a similar background. Interestingly, asymmetries in processing different affixations are also present in word recognition in human. In human linguistic studies, it has been suggested that there is a preference for suffixation in natural languages (Bybee et al. 1990; Cutler et al. 1985; Dryer 2005). Among the various hypotheses offered to explain the suffixation preference is the idea that a suffix does not present a problem for making word recognition more difficult while a prefix does (Dryer 2005). The beginning of a word may hence be its most salient part (Clark 1991) and important for spoken word activation (Marslen-Wilson 1987; Rodd 2004). In the prefixed word, the processing of the stem does not precede the affix, so it is more difficult to do an online processing of the information of the whole word (Kandel et al. 2012). The suffixation preference in language is proposed to be driven by a cognitive mechanism that is not specific to language (Hupp et al. 2009). Our results also demonstrate that biases in processing affixations may be present independently of having language.

To conclude, even though non-human animals lack the syntactic abilities characteristic for language, our results show that they do have the ability to learn about surface transformations similar to affixation patterns and support the hypothesis that such positional learning mechanisms may have been co-opted in human language evolution.
